# Receptor Guanylyl Cyclase C and Cyclic GMP in Health and Disease: Perspectives and Therapeutic Opportunities

**DOI:** 10.3389/fendo.2022.911459

**Published:** 2022-06-29

**Authors:** Hari Prasad, John Kandam Kulathu Mathew, Sandhya S. Visweswariah

**Affiliations:** ^1^ Department of Molecular Reproduction, Development and Genetics, Indian Institute of Science, Bengaluru, India; ^2^ Centre for Biosystems Science and Engineering, Indian Institute of Science, Bengaluru, India

**Keywords:** cGMP (cyclic GMP), guanylyl cyclase C, guanylyl cyclase C agonists, intestine, colorectal cancer type

## Abstract

Receptor Guanylyl Cyclase C (GC-C) was initially characterized as an important regulator of intestinal fluid and ion homeostasis. Recent findings demonstrate that GC-C is also causally linked to intestinal inflammation, dysbiosis, and tumorigenesis. These advances have been fueled in part by identifying mutations or changes in gene expression in GC-C or its ligands, that disrupt the delicate balance of intracellular cGMP levels and are associated with a wide range of clinical phenotypes. In this review, we highlight aspects of the current knowledge of the GC-C signaling pathway in homeostasis and disease, emphasizing recent advances in the field. The review summarizes extra gastrointestinal functions for GC-C signaling, such as appetite control, energy expenditure, visceral nociception, and behavioral processes. Recent research has expanded the homeostatic role of GC-C and implicated it in regulating the ion-microbiome-immune axis, which acts as a mechanistic driver in inflammatory bowel disease. The development of transgenic and knockout mouse models allowed for in-depth studies of GC-C and its relationship to whole-animal physiology. A deeper understanding of the various aspects of GC-C biology and their relationships with pathologies such as inflammatory bowel disease, colorectal cancer, and obesity can be leveraged to devise novel therapeutics.

## Introduction

Beginning in the 1970s, over a decade of research aimed at identifying the receptor for *Escherichia coli* heat-stable enterotoxin (ST) in intestinal epithelial cells led to the cloning and characterization of the receptor guanylyl cyclase C (GC-C) (1–5). Shortly after that, elegant studies provided a detailed characterization of multiple aspects of GC-C biology, including descriptions of its endogenous ligands, downstream signaling, actions in the gastrointestinal system, and potential extra-intestinal effects (6, 7). Although the role of GC-C in the gut remains a substantial focus of basic and translational research, there has been interest in the extra-intestinal functions of the GC-C/cGMP axis (8, 9). These studies, along with the discovery of disease-causing mutations, helped elucidate how GC-C activation and deficiency contribute to human disease (6, 10–12). In the last five years, new and unexplored interactions between GC-C, mucosal homeostasis, the gut microbiome, and host immunity have emerged, along with a better mechanistic understanding of its role in colorectal cancer (13–15).

The identification of mutations or changes in gene expression that perturb the delicate balance of intracellular cGMP levels and fluid and ion homeostasis has opened up new avenues of investigation into the role of GC-C in human health and disease, potentially leading to the development of future therapeutics (6, 7). Except for diarrhea, fluid and ion transport as a mechanistic driver and upstream regulator of intestinal pathologies *per se* has not been the main subject of investigation. Mutations in GC-C that cause impaired intestinal sodium transport have recently brought this theme to the forefront as a key player in altering the composition of the gut microbiome, which in turn orchestrates mucosal immune responses and the development of pathologies such as inflammatory bowel disease (16). An entirely new appreciation has emerged for the intestinal ion-microbiome-immune axis in human health and disease, previously given short shrift due to a lack of causal genetic evidence (16).

**Figure 1:**
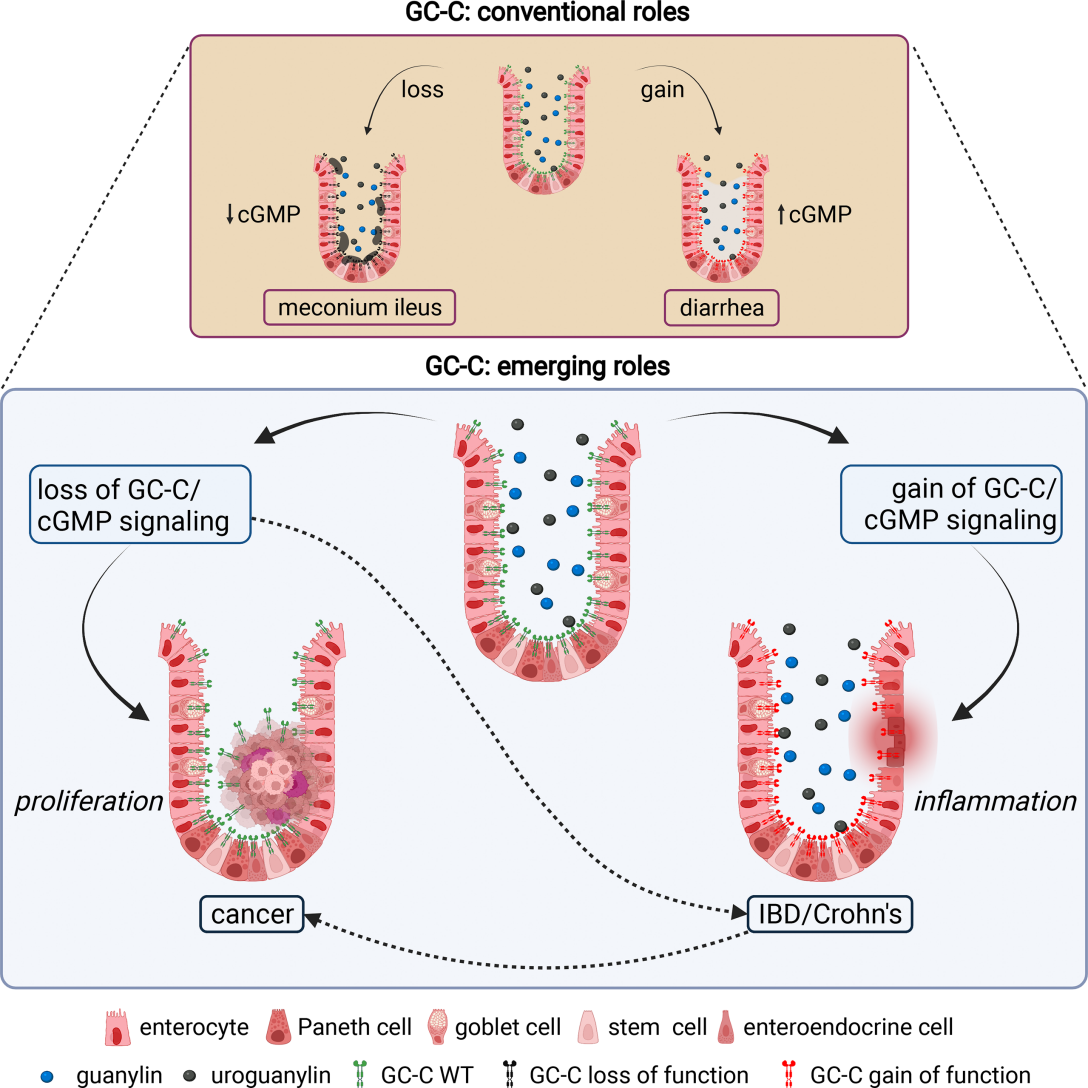
Canonical and emerging roles of GC-C in intestinal homeostasis. The conventional roles of GC-C in the regulation of intestinal fluid ion homeostasis and associated human pathologies are well established. Homozygous and compound heterozygous loss of function mutations in GC-C cause meconium ileus due to decreased fluid and ion secretion. Gain of function mutations in GC-C cause congenital secretory diarrhea due to increased fluid and ion secretion. The emerging roles of the GC-C/cGMP signaling axis in the pathogenesis of several human diseases, most notably colorectal cancer and inflammatory bowel disease, are becoming evident. Loss of GC-C/cGMP signaling because of prominent downregulation of guanylin and uroguanylin is associated with tumorigenesis. Gain of GC-C/cGMP signaling upregulates interferon-stimulated genes and STAT1 activation in intestinal tissue, leading to chronic inflammation and inflammatory bowel disease. Impaired gut barrier integrity and dysbiosis of the microbiome associated with loss (or gain) of GC-C mediated signaling may be linked to inflammatory bowel disease (or colorectal cancer) are shown in dotted lines. The figure was prepared using Biorender.

Although GC-C is highly expressed in the intestine, studies have shown that it is also expressed in extraintestinal tissues, albeit at a much lower level (17–19). It is important to note that the extraintestinal tissues are not generally exposed to the bacterial ST toxin, but the endogenous hormones guanylin and uroguanylin may stimulate the GC-C receptor or function in a GC-C independent manner (9). In this review, we discuss the canonical roles and stimuli of GC-C, extraintestinal effects, and how changes in GC-C-mediated signaling underpin colorectal cancer (CRC) and inflammatory bowel disease (IBD) (**Figure 1**). We describe the development of knockout and transgenic mouse models to study GC-C mediated signaling that integrate physiology, metabolism, and the gut microbiome. We also present our perspectives on how the loss and gain of GC-C mediated signaling may be linked to CRC and IBD and how these seemingly disparate conditions may help broaden our understanding of the cellular roles of GC-C/cGMP signaling in health and disease.

## Canonical Roles of GC-C in the Intestine

### GC-C/cGMP Signaling Pathway

**Figure 2:**
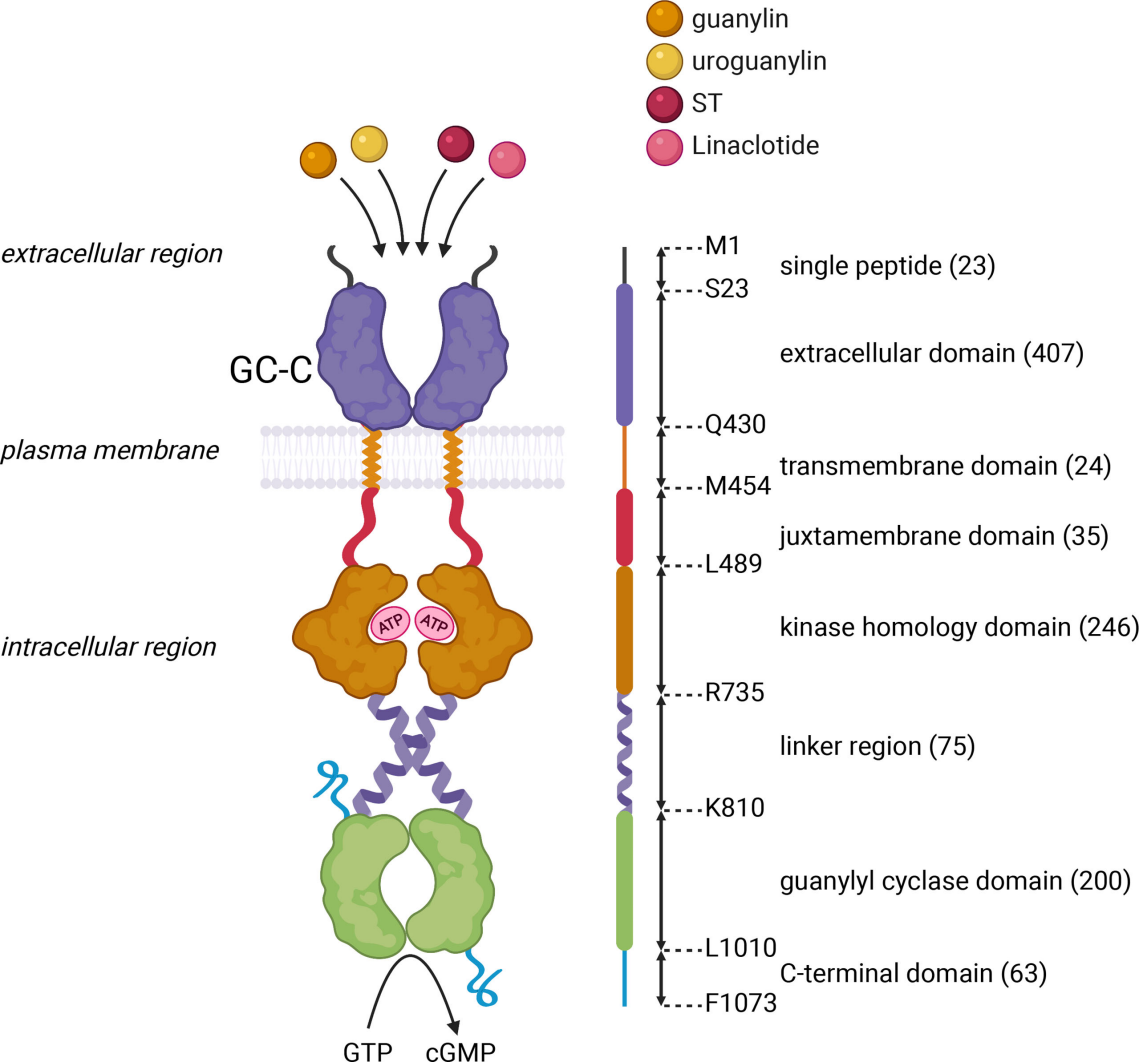
Schematic representation of the domain architecture of GC-C. GC-C is predicted to be a homodimeric multidomain protein that includes an extracellular domain that binds peptide ligands, a transmembrane domain, a juxta-membrane domain, a kinase homology domain that binds ATP, a linker region, a guanylyl cyclase domain that forms a head-to-tail dimer, that converts GTP to cGMP, and a C-terminal domain. The domain boundaries of human GC-C are shown in the linear schematic on the right of the domain architecture, with a single letter amino acid code at each position. Numbers in brackets represent the number of amino acids within the predicted domain boundaries. The figure was prepared using Biorender.

The canonical GC-C/cGMP pathway is well documented as a critical signaling pathway in intestinal fluid and ion homeostasis. GC-C was initially identified as the receptor for a heat-stable enterotoxin (ST) produced by pathogenic enterotoxigenic *Escherichia coli* (ETEC) (3). This transmembrane receptor is encoded by *GUCY2C*. Its predominant expression on the intestinal brush border epithelium (20) strategically positions it to critically regulate fluid ion homeostasis in the gut (13). GC-C is a multidomain protein that includes an extracellular domain (ECD), transmembrane domain, juxta-membrane domain, kinase homology domain (KHD), a linker region, guanylyl cyclase domain (GCD), and C-terminal domain (**Figure 2**). The crystal structure of the GC-C has not yet been solved, but based on our experimental mutagenesis data, which has recently been corroborated by structural studies of the related soluble guanylate cyclase (sGC) protein, we proposed a model that the structural re-arrangement of ECD and KHD, as well as the conformational switch of the linker region, re-arrange the two cyclase domains into an active conformation, enabling catalysis and cGMP synthesis (21–24). The functional and clinical relevance of each domain has recently been reviewed (6).

In 1978, Hughes et al. and Field et al. independently reported that ST increases cGMP by activating GC-C in intestinal epithelial cells, resulting in fluid-ion expulsion from cells and watery diarrhea (25, 26). The supposition that ST produced by ETEC acts as a molecular mimic of endogenous peptides led to the discovery of guanylin and uroguanylin and insight into their mechanisms of action. Guanylin, purified from the rat jejunum, has a high degree of homology with ST, and was shown to increase intracellular cGMP in T84 human colon cancer cells (27). Shortly after, uroguanylin was isolated from opossum urine and intestinal mucosa and named after its natural source and similarity to its predecessor (28). Uroguanylin was also isolated and characterized from human urine (29). These ligands, like many hormones, are secreted by intestinal epithelial cells in their precursor forms (30). Their biologically active forms are C-terminal fragments derived from longer prohormones. The enzymes and mechanisms responsible for the conversion of precursor hormones to active hormones are unknown (31). Furthermore, prouroguanylin and proguanylin, which are constitutively secreted into the gut lumen primarily by mature enterocytes, are released into the bloodstream and can be detected in plasma (31, 32). While GC-C was found to be expressed in intestinal epithelial cells relatively uniformly along the rostrocaudal axis, its ligands were found to have differential expression, with uroguanylin primarily expressed in the small intestine and guanylin primarily expressed in the distal small intestine and colon (31, 33–35). The cellular sources of endogenous GC-C ligands are not well defined, but studies have shown that they are expressed in a variety of cell types, including Paneth cells, goblet cells, entero/colonocytes, enteroendocrine cells, and tuft cells (31, 33–35). Although synthesized uroguanylin was 10-fold more potent than guanylin, neither peptide achieves the potency of ST in activating GC-C. Purified forms of these ligands elicited GC-C mediated intracellular cGMP production (36). Guanylin peptides produced in the gastrointestinal (GI) tract presumably enter the circulatory system, reaching various extraintestinal tissues where the precursor hormones are processed into active forms, bind to GC-C, and modulate the function of the target organ. In addition, guanylin peptides may be produced locally in target organs, have auto/paracrine functions, and contribute to the circulating pool (37).

Multiple lines of evidence suggest that guanylin peptides are luminally secreted in the intestine (38, 39). More recent studies using murine jejunum and colon preparations mounted in Ussing chambers have provided experimental evidence for a secretory pathway for guanylin peptides in the basolateral direction from enterocytes. Notably, while proguanylin peptide has been shown to be released in both the apical and basolateral directions, apical secretion is greater (31). The physiological roles of circulating prohormones, as well as the stimulus and regulation of secretion of these precursors from gut epithelial cells into the intestinal lumen and blood, remain unexplored.

**Figure 3:**
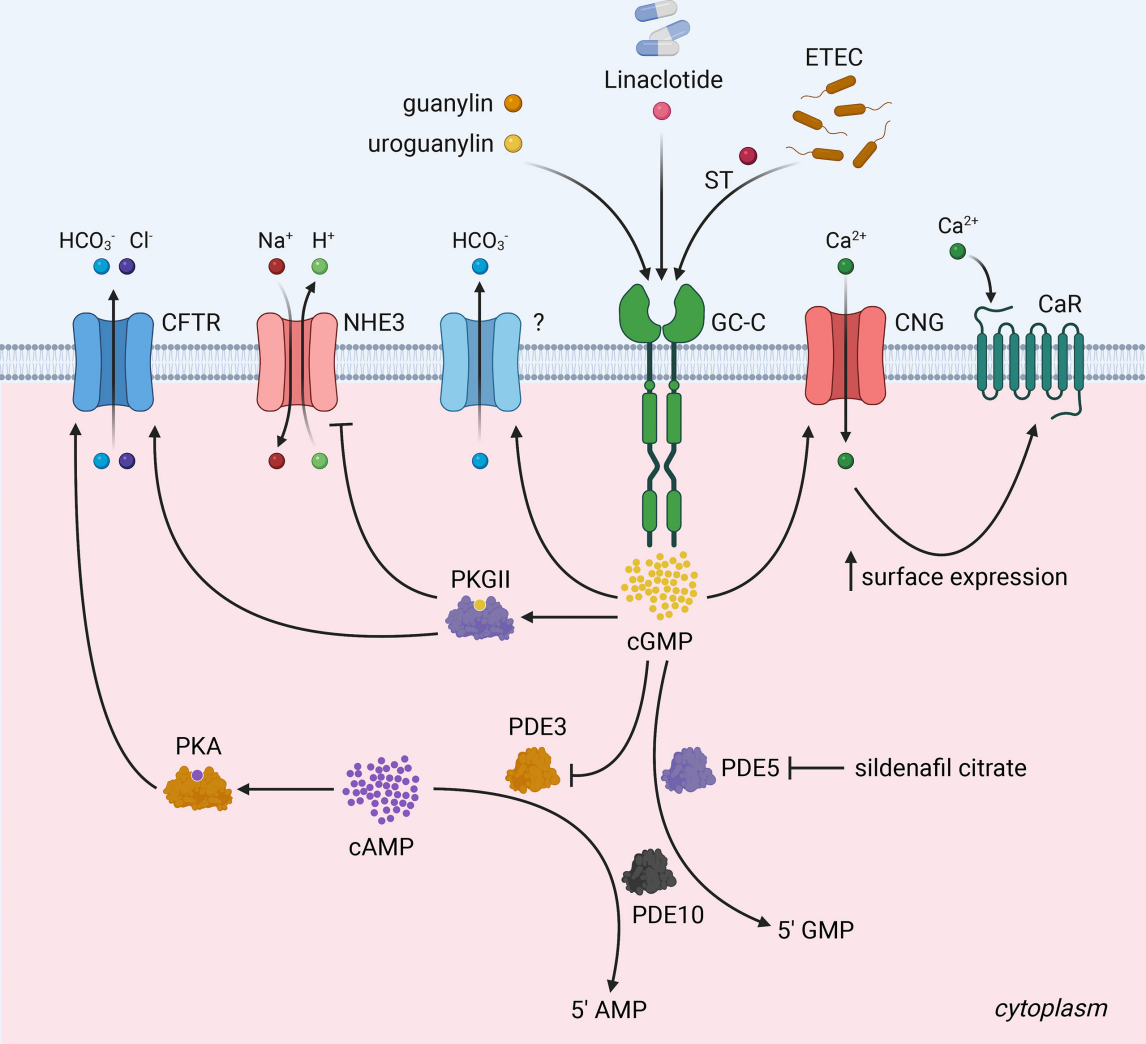
The GC-C/cGMP signaling axis and fluid-ion homeostasis in the intestine. Binding of ligands (heat-stable enterotoxin/ST) produced by enterotoxigenic *E.coli* and the endogenous hormones guanylin and uroguanylin) to GC-C catalyzes the formation of cGMP from GTP. Increased intracellular cGMP levels result in the activation of cGMP-dependent protein kinase II (PKGII) and the inhibition of cAMP-specific phosphodiesterase (PDE3), which in turn leads to the cross-activation of cAMP-dependent protein kinase (PKA). Reduced intestinal sodium absorption is caused by PKGII-mediated inhibitory phosphorylation of NHE3. PKGII and PKA phosphorylated and activate the cystic fibrosis transmembrane conductance regulator (CFTR) anion channel, increasing intestinal chloride and water secretion. Elevated intracellular cGMP increases duodenal bicarbonate secretion *via* CFTR and perhaps additional unknown mechanisms. Increased cGMP activates cyclic nucleotide-gated ion channels (CNG), promoting Ca^2+^-influx, which recruits calcium-sensing G-protein coupled receptors (CaR) to the plasma membrane. PDE5, a cGMP-dependent phosphodiesterase, and PDE10 hydrolyze cGMP to 5’ GMP to attenuate GC-C signaling. Pharmacological GC-C agonists (e.g., linaclotide) and cGMP-specific phosphodiesterase PDE5 antagonists (e.g., sildenafil citrate) increase intracellular cGMP levels suggesting, that they may have a synergistic antiproliferative effect and reduce the likelihood of resistance to both drugs in colorectal cancer. The figure was prepared using Biorender.

In intestinal epithelial cells, GC-C is a major source of cGMP. Physiologically, ligand binding to GC-C catalyzes cGMP formation from GTP, resulting in the activation of cGMP-dependent protein kinase II (PKGII) (6, 20, 40, 41). PKGII-mediated phosphorylation and consequent inhibition of sodium hydrogen exchanger isoform 3 (NHE3) reduce intestinal sodium absorption (42, 43). Furthermore, elevated intracellular cGMP levels may inhibit cAMP-specific phosphodiesterase (PDE3), leading to an increase in cAMP levels and cross-activation of cAMP-dependent protein kinase (PKA) (44). Both PKGII and PKA can activate the cystic fibrosis transmembrane conductance regulator (CFTR) anion channel, increasing intestinal chloride and water secretion (44, 45) (**Figure 3**). PKGII also phosphorylates vasodilator-stimulated phosphoprotein (VASP), which can be used as a readout of GC-C activation (46). As outlined above, the known biological roles of GC-C are primarily dependent on its ability to produce cGMP. Any cGMP-independent functions of GC-C remain to be determined. The latest understanding in this regard has come from genetic and physiological studies using Gyc76C, a receptor guanylyl ortholog in *Drosophila melanogaster* (47). Gyc76C, the closest ortholog of human GC-C (http://www.flyrnai.org/diopt), regulated humoral responses of fly larvae to bacterial infections in a cGMP-dependent manner, while cellular responses were cGMP-independent, and did not require the GCD but required a functional KHD (47).

### Mouse Models to Study GC-C/cGMP Axis

Mutations in the *GUCY2C* gene may result in meconium ileus due to loss of function (12) or familial diarrhea due to gain of function (10, 11) (**Figure 1**). The development of animal models to study GC-C/cGMP signaling and mimic human pathophysiology associated with these mutations is critical for biomedical research and diarrheal disease studies, as well as being a useful tool for discovering therapeutic drug targets in preclinical studies. The gene encoding GC-C (*Gucy2c*) is mapped to chromosome 6 in mice and chromosome 12p12 in humans. Two independent groups developed GC-C knockout mice that were found to be viable and showed no apparent changes in intestinal fluidity (48, 49). Studies in these GC-C knockout mice have revealed that GC-C/cGMP signaling is required for the mediation of ST-induced diarrhea, protection against enteric pathogens, and the maintenance of microbiota homeostasis (14, 48–50). GC-C knockout mice also displayed extraintestinal phenotypes such as adipose mass hypertrophy and steatohepatitis, exacerbating the metabolic syndrome associated with diet-induced obesity, such as cardiac hypertrophy and impaired glycemic control (51).

Mouse models with deletions of guanylin (52) and uroguanylin (53) have been created. Guanylin null mice were viable with no intestinal obstruction or malabsorption. These mice had lower levels of cGMP in the colonic epithelia, which correlated with a significant increase in the rate of colonic epithelial proliferation and an accelerated turnover of cells along the crypt-villus axis, while the amount of apoptosis remained unchanged (52). On the other hand, uroguanylin null mice had lower cGMP levels in the small intestine, but fluid-ion homeostasis in the gut appeared to be maintained (53). Interestingly, these mice had increased blood pressure and an impaired natriuretic response to dietary salt intake, indicating that uroguanylin plays a role in maintaining overall salt homeostasis in the body (53).

Recently, a transgenic mouse model was developed and studied to decipher the cellular origins of guanylin using fluorescent reporter (Venus) expression driven by the proguanylin promoter (31). Proguanylin-expressing cells were found throughout the small intestine and colon but were scarce in the duodenum (31). Additionally, transgenic mice with the EGFP reporter gene inserted immediately upstream of the coding sequence of the *gucy2c* gene generated by the GENSAT Project and made available by the Mutant Mouse Regional Resource Center (https://www.mmrrc.org/catalog/sds.php?mmrrc_id=30480) could be a useful resource for studies defining its expression, localization, and function in intestinal, neuronal, and other cell types.

We recently reported a novel mouse model with an activating mutation in *Gucy2c* (p.Ser839Ile) equivalent to that seen in an affected Norwegian family with familial diarrhea syndrome (p.Ser840Ile) (13). As anticipated, these mice showed elevated cGMP levels in the small intestine and diarrhea-like features, including increased fecal sodium and water content (13). Significantly, our findings in this mutant mouse model demonstrating dysbiosis and increased susceptibility to dextran sulfate sodium (DSS)-induced colitis delineated an essential rheostatic role of GC-C signaling in intestinal homeostasis (13).

## Single-Cell Dissection of GC-C/cGMP Axis in the Intestine

Single-cell analysis technologies have advanced our understanding of human disease by enabling unbiased and comprehensive analysis of cellular diversity within a tissue (54). Multiple studies have used single-cell sequencing to profile intestinal epithelial cells, identify novel subtypes, characterize gene signatures, assess clonal evolution of tumor cell lineages, and gain insights into somatic mutations and drug response (54, 55). A study employing human colonic cell single-cell RNA sequencing to uncover epithelial cell diversity in health and IBD discovered a novel pH-sensing absorptive colonocyte named BEST4/OTOP2 cells, which have distinct expression of the calcium-sensitive chloride channel BEST4 and the proton-selective channel OTOP2 (56). BEST4 expression is predicted to mark colonic epithelia involved in salt, ion, and metal transport. Intriguingly, the cells in this cluster expressed mature colonocyte markers, most notably the genes encoding uroguanylin (*GUCA2B*) and guanylin (*GUCA2A*). Physiologically, the high expression of guanylins in BEST4/OTOP2 cells at the top of the crypts may aid in pH sensing and the maintenance of luminal homeostasis *via* regulation of the GC-C/cGMP signaling pathway. Indeed, functional studies have revealed that these specialized cells conduct protons into the cytoplasm in response to extracellular acidification, resulting in significant acidification of intracellular pH. Further, BEST4/OTOP2 cells have high levels of the anti-apoptotic protein BAG1, which may allow them to survive substantial pH changes (56).

An emerging understanding is that the unique acidic environment created within the BEST4/OTOP2 cell in response to extracellular acidification may be required for downstream functions. Notably, intracellular acidification is a well-known trigger for activating NHE3, which transports protons out of cells in exchange for Na^+^ ions, thereby inhibiting excess proton accumulation in intestinal epithelial cells (16, 57, 58). We can speculate that the mechanistic role of guanylins and GC-C/cGMP signaling in BEST4/OTOP2 cells is to inhibit NHE3 and prevent the leak of protons from the cytosol imported in response to extracellular acidification, thereby facilitating the build-up of protons that initiates downstream signaling. We further note that intracolonic pH is relatively acidic and ranges between 5 and 7 along the human colon, depending on the type and abundance of gut microorganisms and their fermentation products and bicarbonate secretion by colonic epithelial cells (59, 60). Experimental observations show that when the extracellular pH was 7, there was no significant difference in intracellular pH between BEST4+ and BEST4- cells, but at pH 5, there was substantial acidification (∼0.5 pH unit lower) of the cytoplasm in BEST4+ cells compared to BEST4- cells (56). BEST4/OTOP2 cells may sense pH changes caused by microbiota composition, regulate host-microbiome interactions, and control mucosal immunity. Further studies are needed to substantiate these hypotheses.

Recently, single-cell analysis in human biopsy specimens also identified a rare BEST4+ cell type in the duodenum (~1.3% of all epithelial cells), which may play a role in maintaining normal fluid ion homeostasis (61). Importantly, BEST4+ cells in the duodenum, like those in the colon, express high levels of the *GUCA2B* and *GUCA2A* genes (61). GC-C was found to be ubiquitously expressed in the duodenal epithelium. Furthermore, because BEST4+ cells have the highest level of CFTR expression among all duodenal cells, they are referred to as BEST4, CFTR high-expressor (BCHE) cells (61). While *GUCA2B* and *GUCA2A* expression are specific to BCHE cells in the duodenum, they are broadly expressed in many colonic enterocytes. Additionally, CFTR expression was found in BEST4+ cells in the duodenum but not in the colon, implying that BEST4+ cells in the colon and BEST4+ cells in the duodenum may serve different functions. Selective expression of guanylin and uroguanylin in BEST4+ cells in the duodenum would result in increased GC-C/cGMP signaling and downstream constitutive activation of CFTR, which may mediate the high-volume fluid secretion required to neutralize the acidity of chyme from the stomach (61).

Several recent single-cell studies have provided novel insights into the role of the GC-C/cGMP signaling axis in various human pathologies. For example, a single-cell analysis to determine the importance of aryl hydrocarbon receptor (Ahr)-dependent signaling in shaping cellular differentiation potency and suppressing colon tumorigenesis discovered that Ahr deletion changes the landscape of colonic crypt cell-cell communication, including the complete loss of the GC-C/cGMP pathway (62). Another intriguing study found a distinct population of enterocytes (enterocytes 1) in the colon- and ileum-derived human organoids that specifically expresses *GUCA2A* but is not susceptible to SARS-CoV-2 infection despite high levels of angiotensin-converting enzyme 2 (ACE2), the cellular receptor of SARS-CoV-2-mediating viral entry (63). Increased expression of interferon-stimulated genes (ISGs) in these cells may underpin their resistance to SARS-CoV-2 infection (63). It is possible that GC-C/cGMP signaling modulates the cell-intrinsic innate immune response to inhibit virus replication and spread, based on findings (13).

## GC-C and Colorectal Cancer: Roles in Tumorigenesis and Clinical Potential

### Multi-Dimensional GC-C-Mediated Regulation of Cytostasis

Periodic tides of cell regeneration replace the older cells along the crypt-villus axis *via* cytostatic regulation mediated in part by the GC-C/cGMP pathway (64). Despite repeated assaults on the gut, a normal and healthy epithelial layer is maintained (65). GC-C regulates stem cells at the base of intestinal crypts, which migrate and differentiate to form enterocytes and other cell types (46, 65).

**Figure 4:**
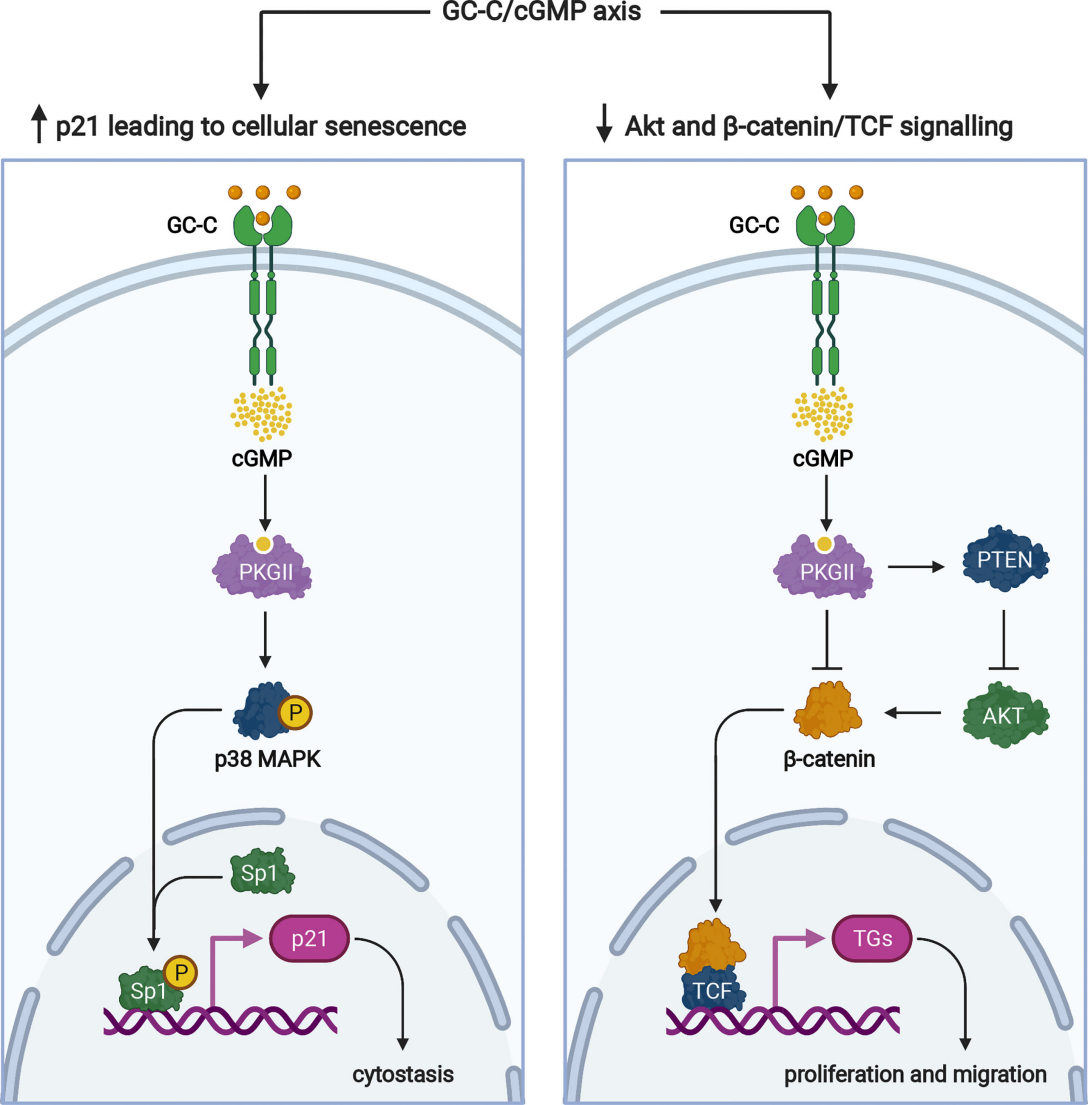
Signaling pathways of the GC-C/cGMP axis that regulate cellular proliferation. Ligand-mediated activation of GC-C increases intracellular cGMP. Cyclic GMP production activates PKGII and p38 MAPK resulting in phosphorylation of the Sp1 transcription factor. Sp1 upregulates the expression of p21 and mediates cytostasis. PKGII-mediated signaling opposes pro-survival and pro-proliferative phenotypes mediated by the β-catenin/TCF and Akt pathways. Increased GMP activates cyclic nucleotide-gated ion channels (CNG), promoting Ca^2+^-influx that mediates cytostasis and recruiting calcium-sensing G-protein coupled receptors (CaR) to the plasma membrane. TGs denotes target genes. The figure was prepared using Biorender.

Multiple proteins regulate cell cycle progression, one of which is the cyclin-dependent kinase inhibitor, p21. We demonstrated that GC-C/cGMP signaling increases Sp1-mediated p21 transcription in colonic cancer cells, induces cellular senescence, and has antitumorigenic properties (64) (**Figure 4**). Because GC-C is a substrate for inhibitory phosphorylation by c-src tyrosine kinase (66), this cytostasis mechanism is controlled by cross-talk between GC-C and c-src. Under normal physiologic conditions, GC-C regulates basal cGMP levels and the delicate balance between proliferation and differentiation through its downstream effects mediated by increased p21 expression (64).

In addition to p21-mediated cytostasis, several GC-C-mediated antitumorigenic mechanisms have been described. The most notable example is GC-C/cGMP signaling-mediated attenuation of β-catenin-mediated TCF transcriptional activity (67, 68) (**Figure 4**). PKGII-mediated signaling opposes pro-proliferative and pro-migratory phenotypes mediated by β-catenin/TCF (67, 68). In turn, β-catenin/TCF signaling dampens the GC-C axis by silencing the transcription of its ligands, guanylin and uroguanylin (15). GC-C/cGMP has also been shown to inhibit protumorigenic Akt signaling *via* a PTEN-mediated mechanism (69). Furthermore, because Akt can increase β-catenin nuclear accumulation by directly phosphorylating β-catenin or indirectly stabilizing β-catenin through inhibition of GSK-3β, inhibition of Akt signaling may also be involved in GC-C-mediated attenuation of β-catenin/TCF transcriptional activity (70) (**Figure 4**).

Both apoptosis and autophagy are regulated by Akt signaling (71). Akt promotes cell survival by inhibiting pro-apoptotic signals from Forkhead box O (FOXO) transcription factors. FOXO regulates cell survival by either directly targeting cyclin-dependent kinase inhibitors such as p21 and p27, or indirectly, by influencing cyclin D1 and p53 (72). Early stages of colon tumorigenesis are characterized by GC-C signaling attenuation, which is associated with the over-activation of Akt, a common integrator of mitogenic, pro-oncogenic, and tumor suppressor signals, putting GC-C at the crossroads of homeostasis and tumorigenesis (69). Ligand-mediated activation of GC-C in colorectal cancer cells replenishes cGMP and specifically downregulates Akt without affecting mitogenic-activated protein (MAP) kinase pathways (69).

GC-C-mediated regulation of intracellular calcium levels and cytostasis has been described (73). Increased intracellular cGMP activates cyclic nucleotide-gated ion channels (CNG), promoting Ca^2+^-influx, which recruits calcium-sensing G-protein coupled receptors (CaR) to the plasma membrane (73) (**Figure 3**). Taken together, the regulation of cytostasis by GC-C mediated signaling *via* several pathways is important in understanding the role of GC-C in carcinogenesis. Further research into the interplay of such regulatory mechanisms as “cytostasis determinants” will most likely shed light on the emerging role of GC-C/cGMP signaling in colorectal cancer (**Figure 1**).

### GC-C and Its Ligands in Pre-Cancerous Lesions

Epidemiologic studies have found a link between countries prone to enterotoxigenic *E.coli* infections and a low prevalence of colorectal cancer. Although this is far from indicating causation, such a link may imply that repeated exposure to ST has some beneficial effects and an anti-proliferative role in the early stages of cancer (74). Allelic imbalance is seen in early colorectal cancers on chromosome 1p (75), where the genes encoding guanylin and uroguanylin are located. As previously discussed, guanylin expression varies along the duodenal-to-colonic axis of the intestine, with maximum expression in the distal small intestine and colon (76). In contrast, uroguanylin is predominantly expressed in the small intestine (33, 77). Because uroguanylin and guanylin expression is negatively regulated by Wnt signaling, they are mostly found on the surface epithelium facing the lumen, which has tapering Wnt expression, as opposed to GC-C, which is expressed along the crypt-to-surface axis (15). Most colorectal tumors are caused by adenomatous polyposis coli (APC) loss-of-function mutations or β-catenin gain-of-function mutations, both of which result in abnormal Wnt signaling activation (78). This is consistent with a substantial reduction in guanylin and uroguanylin expression during the early stages of tumorigenesis in colorectal cancer compared to normal tissue (15) (**Figure 1**). The loss of GC-C ligands early in the transformation process suggests that oncogenic pathways disrupt the normal cellular homeostasis maintained by GC-C. Can these ligands be used as colorectal cancer diagnostic or prognostic markers? This is an important question to consider.

Aberrant crypt foci (ACF) are pockets of hyperproliferation seen in cancer biopsies and are classified as premalignant lesions (79). Treatment with methyl-N-nitrosourea (MNU) increases ACF formation in GC-C null mice compared to WT. Furthermore, ST or cGMP analog treatment failed to reverse ACF in GC-C null mice (64). Studies in the Apc^Min/+^ mouse, which carries an inactivated allele of the *Apc* gene, also demonstrated that activation of GC-C protects against the development of multiple polyps (65, 80). Even though guanylin and uroguanylin are gene products that are lost early in colorectal carcinogenesis, GC-C expression is maintained in the majority of colorectal cancers. This has sparked interest in studies to see if uroguanylin-mimetics can be used as a chemoprophylactic drug for people at high risk of developing colorectal cancer (65, 80). Importantly, because guanylin and uroguanylin expression is restricted to normal regions of the intestine, an *in vivo* tracking technology for these peptides could be used to differentiate cancer-affected parts of the intestine from the rest of the intestine.

### GC-C Ligand-Mediated Anti-Cancer Therapies

Several GC-C agonists have completed clinical trials. Although linaclotide, a 14-amino acid uroguanylin mimetic, has been used to treat chronic constipation and irritable bowel syndrome, the FDA-approved formulation of oral linaclotide developed for small-bowel delivery was found to be insufficient for inducing GC-C in the colorectum and preventing tumorigenesis in humans (81). In preclinical studies, linaclotide inhibited tumorigenesis and polyp formation in Apc^Min/+^ mice when compared to an untreated control group (82). Another GC-C agonist, plecanatide, showed an anti-proliferative effect in Apc^Min/+^ mice *via* GC-C/cGMP signaling, due to suppression of the Wnt/β-catenin pathway as well as other pro-inflammatory cytokines (83). Dolcanatide, a uroguanylin mimetic with improved stability, has been developed and unlike other GC-C agonists, is resistant to proteolysis in the intestinal milieu. Daily administration of this drug has been shown to improve the condition of DSS-induced colitis in mice by activating GC-C/cGMP signaling (84). However, this is in contrast to the recent study using transgenic gain-of-function mutant mice which showed a greater susceptibility to DSS (13).

GC-C agonists as monotherapy may increase the risk of tumor cells developing resistance to these drugs. This is because chronic GC-C stimulation may induce cGMP-specific phosphodiesterase PDE5 (85, 86), which lowers cGMP levels and impairs Ca^2+^ influx through CNG channels (73). Phosphodiesterase inhibitors prevent cGMP degradation (87). Sildenafil is a PDE5 inhibitor drug approved for erectile dysfunction (ED) (88), but studies in mice have repurposed it to target colorectal cancers (87, 89). Sildenafil treatment effectively reduces polyp formation in APC^Min/+^ mice (82) and prevents inflammation-induced tumors in AOM/DSS mice model (90). Vardenafil, a similar drug, raises cGMP levels in the colonic mucosa of wild-type mice and is thought to have chemo-preventive properties (91). Since it causes a cGMP-mediated increase in PKGII activity, it inhibits proliferation and apoptosis without negatively affecting differentiation in the colonic epithelium (92, 93). Additionally, PDE5 inhibitors could prevent tumor formation by stabilizing cGMP levels and improving epithelial barrier function (94). PDE5 antagonists, like GC-C agonists, increased luminal apoptosis to maintain cellular homeostasis and suppressed tumor proliferation (82). PDE10, another cGMP-degrading phosphodiesterase, is also overexpressed in colon cancer cells compared to normal colonocytes (67). Indeed, PDE10 could be a potential mechanism conferring resistance to PDE5 inhibitors (82). Thus, combining two drugs, GC-C agonists and PDE5 antagonists, which both increase intracellular cyclic GMP levels *via* complementary mechanisms of action, may result in a synergistic antiproliferative effect that reduces the likelihood of resistance to both drugs (**Figure 3**).

### Emerging GC-C-Directed Colorectal Cancer Therapies

In recent years, chimeric antigen receptor T (CAR-T) cells, which involve engineering patients’ immune cells to treat cancer, have sparked considerable interest among scientists and oncologists. Despite a few setbacks, CAR-T therapy has demonstrated promising therapeutic efficacy in treating hematological malignancies (95–97). The specific targeting of the CD19 cell marker is critical to the success of this therapy in hematological malignancies (98). In comparison, the development of CAR-T cell therapy for solid tumors such as colorectal cancer has stalled due to a lack of tumour-specific antigens.

In this regard, GC-C has emerged as an attractive candidate for CAR-T cell therapy in colorectal cancer for the following reasons: first, the broad selectivity of GC-C expression in the human intestine; second, expression on the cell surface; and finally, the ability to maintain expression in colorectal cancers at both primary and metastatic sites (99). Preclinical studies in mice models demonstrated that GC-C-targeted CAR-T cells effectively induce T-cell activation and effector function, recognize and kill human colorectal cancer cells without toxicity or autoimmunity, and provide protection against metastatic colorectal cancer (100, 101). CAR-T-based approaches targeting GC-C have entered clinical trials (https://clinicaltrials.gov/ct2/show/NCT04652219). Furthermore, while CAR-T cells targeting GC-C in colorectal cancer may be promising due to the gut-specificity of GC-C expression and its universal expression in primary tumors and distant metastases, these approaches may also be promising in targeting tumors arising from intestinal metaplasia, such as esophageal, gastric, and pancreatic cancers with ectopic GC-C expression (99, 102, 103).

Other emerging GC-C-directed modalities for the early diagnosis and treatment of colorectal cancer include photodynamic diagnosis (PDD) and photodynamic therapy (PDT), which are light-based approaches that can be used for the early diagnosis and treatment of colorectal cancer (104). Recently, PDD and PDT approaches involving zinc phthalocyanine as a photosensitizer bound to a polyethylene glycol-gold nanoparticle that could be directly delivered to a colorectal cancer site using a specific antibody against GC-C were developed and tested in cell culture studies (105, 106).

## Regulatory Role of GC-C in Intestinal Inflammation and IBD Pathology

Inflammatory bowel disease (IBD) is becoming more common. Historically, it was a condition of the wealthy, but it is now fast spreading to developing countries (107, 108) with an increasing trend in the juvenile age group (109). Two subtypes of IBD are Crohn’s disease (CD) and ulcerative colitis (UC). The former can affect any part of the gastrointestinal tract, while the latter affects the colon and rectum (110). Although our understanding of the disease and its response to treatment has progressed (111), we still have a long way to go in uncovering the mechanistic drivers in the pathogenesis of IBD. Both the immune system and the microbiota have co-evolved and shape gut homeostasis, which has far-reaching effects on other organ systems (112). There is a growing recognition that IBD is a complex disease with an interplay of incompletely defined genetic and environmental risk factors that disrupt the microbiome-immune axis (110). Research into rare monogenic causes of IBD may shed light on common sporadic adult-onset disease by identifying pathways that act as upstream pathogenic factors of disease pathology.

In this context, it is important to note that IBD is associated with GC-C gain of function mutations, implying that activation of the GC-C/cGMP axis could be a key to underlying IBD predisposition. One of the earliest insights linking GC-C and IBD was provided by the study of patients with familial diarrhea syndrome due to an activating mutation in GC-C (S840I) in one large Norwegian kindred, more than 25% of whom were diagnosed with Crohn’s disease (10). More recently, mutations in GC-C have been identified as a common monogenic cause of pediatric-onset IBD (113). The mutational spectrum of GC-C and associated human phenotypes, including IBD has recently been reviewed elsewhere (6, 16). Given the relatively small number of activating mutations associated with GC-C reported in the literature, definitive genotype-phenotype correlations are not possible. However, two mutations in the GC-C linker region with the highest cGMP levels had more severe complications, including early-onset IBD (11).

Loss of function of NHE3, a well-known effector, negatively regulated by GC-C, has also been linked to IBD-like pathologies in humans and mice models (57, 114, 115), indicating that the impairment of Na^+^ absorption shared by both activating GC-C and inactivating NHE3 mutations could be a pathogenic driver in IBD. In line with this hypothesis, impaired sodium absorption is a long-recognized pathological feature in IBD and microscopic colitis, a poorly understood type of IBD that is nearly as common as CD and UC (16). Several studies have shown reduced NHE3 expression/activity in IBD patients and after exposure to proinflammatory cytokines and *Clostridium difficile* toxin B; however, it has been uncertain until recently whether the downregulation of NHE3 plays a causal role rather than simply reflecting the disease (116–118). The discovery of mutations in GC-C and its downstream effector NHE3 in patients with IBD has highlighted impaired intestinal sodium transport as a key player in altering the composition of the gut microbiome, which in turn orchestrates mucosal immune responses. This ion-microbiome-immune axis will continue to provide critical insight into the pathogenesis of IBD and new avenues for prevention and treatment. Supporting evidence comes from *in vitro* studies demonstrating the ability of high sodium levels and high pH correlating with the intestine of patients with activating mutations in GC-C to promote the growth of a colitogenic pathobiont *Bacteroidetes thetaiotaomicron* (119, 120).

A transgenic mice model was recently developed to study a human mutation in GC-C (S840I) associated with familial diarrhea syndrome with increased susceptibility to IBD (13). Transgenic mice were more vulnerable to DSS-induced colitis than wild-type mice, as evidenced by lower body weight and higher fecal lipocalin levels, indicating a higher disease activity index. Furthermore, transgenic mice with GC-C activation demonstrated prominent gut microbial dysbiosis, emulating the microbiome of IBD and familial diarrheal syndrome patients with S840I mutation in GC-C (121). An increase in opportunistic pathogen species (*Anaeroplasma, Desulfovibrio, Mucispirillum, and Paraprevotella*) and a decrease in protective bacteria (*Colidextribacter, Dorea, Dubosiella, and Lactobacillus*) in transgenic mice were seen, making them vulnerable to environmental factors that promote gastrointestinal inflammation (13). The predicted functional composition of the fecal microbiome revealed that those pathways linked to host immunity and IBD pathogenesis, such as IL17 signaling and Th17 cell differentiation, NOD-like receptor signaling, antigen processing and presentation, were enriched in transgenic mice (13). Interestingly, pathways governing the breakdown of polycyclic aromatic hydrocarbons were downregulated, possibly predisposing to cancer, while chemical carcinogenesis pathways were also downregulated, possibly indicating a protective role against tumorigenesis in transgenic mice with GC-C activation (13).

It is worth noting that GC-C deletion in mice also resulted in gut microbial dysbiosis, which contributed to increased *Salmonella* spp. infection, highlighting a complex relationship between GC-C signaling and the gut microbiome (14). Pathway analysis and experimental validation of transcriptomic data from colonic tissue from transgenic mice with GC-C activation revealed that GC-C/cGMP activation activates STAT1 in ileal and colonic tissue, resulting in increased expression of interferon-stimulated genes and inflammation in the gut (13) (**Figure 1**). In summary, the mice model of activating mutation in GC-C has provided mechanistic understanding of IBD associated with familial diarrhoea syndrome at multiple biological levels ranging from transcriptome, molecular to cellular, and microbiome. Therefore, any disruption in fluid and ion transport may disrupt microbial homeostasis, activate proinflammatory signaling, and promote host-damaging mucosal inflammatory responses.

## Extraintestinal Roles of GC-C/cGMP Signaling

### GC-C at the Crossroads of Gut-Brain Axis, Behavioral Functions, and Visceral Nociception

What controls the balance between energy intake and energy expenditure has been enigmatic for decades. The gut-brain axis has emerged as a critical player in this process (122). Numerous hormones and factors have been implicated in the coordinated control of energy homeostasis, both centrally and peripherally (122). In this context, the GC-C/cGMP system has emerged as the one with the most important functions. While guanylin hormones, which are released after a meal, centrally mediate satiety and energy expenditure, they may also act locally on adipocytes and regulate their function (51, 123, 124). Importantly, recent research has revealed additional functions for the GC-C signaling in the nervous system that go beyond feeding/satiety circuits, such as their roles in behavioral functions and visceral nociception, indicating a more extensive role in controlling neurophysiology (125, 126).

The hypothalamus is the control center for energy homeostasis, and nuclei within the hypothalamus crosstalk, integrate obesogenic and anti-obesity signals, and regulate appetite and energy expenditure (127). The anorexigenic effects of GC-C are reported to be mediated by its expression in the hypothalamic arcuate nucleus (ARC), which contains neuronal populations that communicate with other hypothalamic areas involved in appetite control and play an important role in regulation of energy homeostasis (124, 127). Physiologically, an increase in uroguanylin peptide levels after a meal leads to satiety and energy balance to maintain a homeostatic state (51). Indeed, prouroguanylin/uroguanylin levels in plasma and intestine were found to be lower in obese individuals and, more importantly, do not rise in circulation in response to a meal, which is consistent with obese mice, which had decreased leptin-dependent uroguanylin secretion after a meal (123, 128–130). In line with this idea, intravenous or intraventricular administration of GC-C ligands to mimic an energy-efficient state induced satiety in wild-type mice but not in GC-C null mice and resulted in significant weight loss in diet-induced obese mice, further confirming a crucial anorexigenic role of GC-C signaling (51, 131, 132). Notably, activation of GC-C signaling in hypothalamic neurons increased expression of the anorexigenic neuropeptide proopiomelanocortin, which may activate catabolic pathways, resulting in decreased food intake and increased energy expenditure (51, 127). Furthermore, central uroguanylin administration increased sympathetically innervated brown adipose tissue (BAT) thermogenesis, indicating that central GC-C signaling not only regulates feeding but also energy utilization and fat accretion by modulating sympathetic output, BAT thermogenesis, and browning of white adipose tissue (131). It should be noted that contradictory data has also been published, indicating that neither central nor peripheral administration of GC-C ligands affects food intake or glucose homeostasis (133, 134).

There are two discrete neuronal circuits expressing GC-C reported in the literature, one originating in the tyrosine hydroxylase negative neurons of the ventral pre-mammillary nucleus (PMV) in the hypothalamus and the second in the tyrosine hydroxylase positive neurons of the ventral tegmental area (VTA) and substantia nigra (SN) in the midbrain, both of which independently project to other sites throughout the brain (18). In neurons, GC-C may play a role in ion fluxes across the plasma membrane, coordinating neuronal activity and interactions between neurons (135). Interestingly, recent research has identified two distinct uroguanylin signaling mechanisms in the brain: a GC-C dependent pathway that hyperpolarized neurons (Purkinje cells of the cerebellum) and a GC-C independent pathway that increased calcium levels in astrocytes (136). There are still unanswered questions about the mechanism(s) of action of GC-C in neuronal circuits, how the GC-C system integrates orexigenic and anorexigenic signals to control energy expenditure and energy balance, and how central GC-C signaling modulates peripheral tissue functionality and obesity pathophysiology. Furthermore, research into the role of the peripheral GC-C system in regulating adipocyte function has gained momentum, and aspects of these new roles will be discussed later in this review.

The expression of GC-C in dopamine neurons in the VTA and SN indicates that GC-C plays a diverse role in controlling neurophysiological functions including, but not limited to, satiety and energy homeostasis (18). In line with this notion, electrophysiological studies showed that GC-C activation increased excitatory potentials of midbrain neurons mediated by glutamate and acetylcholine receptors in a cGMP/PKG dependent fashion (125). Importantly, GC-C knockout mice exhibited an attention deficit hyperactivity disorder (ADHD)-like phenotype (125). Consistent with these findings, a human study found a link between single nucleotide polymorphisms in *GUCY2C* and ADHD and its core symptoms (137). However, contradictory data were recently published, indicating that GC-C knockout mice did not exhibit ADHD-like phenotypes but instead displayed cognitive and startle phenotypes (138). This could indicate that other molecules or pathways compensate for the loss of GC-C in midbrain dopamine neurons. More research utilizing neuron-specific deletions is required to shed light on the functional contributions of GC-C signaling in neuropsychiatric disorders associated with midbrain dopamine system malfunctions, such as ADHD, addiction, Parkinson’s disease, and schizophrenia.

Irritable bowel syndrome (IBS) is a functional gastrointestinal pain disorder characterized by an abnormal brain-gut axis, with psychological stress being a risk factor (139). Uroguanylin mimetics have been US Food and Drug Administration (FDA) approved for treatment of constipation associated with IBS (140). Furthermore, these drugs have been demonstrated to have potent antinociceptive effects in preclinical models of visceral hypersensitivity, and thus have been shown to relieve abdominal pain in IBS patients (126, 141). However, unlike the mechanisms ascribed for GC-C in the hypothalamus and midbrain, little is known about pathways regulating the role of GC-C in visceral nociception. The GC-C-mediated regulation of visceral hypersensitivity has been studied using genetic models as well as pharmacological approaches. In preclinical models, GC-C agonism was shown to reduce post-inflammatory visceral hypersensitivity as well as stress-induced visceral hypersensitivity (126, 142). Mechanistically, ligand-mediated activation of GC-C signaling in intestinal epithelial cells was found to release cGMP into the submucosa, where it inhibited nociceptive afferent signaling, or bottom-up sensitization, and mediated analgesic effect (126, 141). Further understanding the physiological role of the GC-C signaling in visceral nociception not only could provide insights into the aetiology of IBS but is also necessary for the design of novel and rational approaches for abdominal pain relief.

### The Potential Role of GC-C in Liver Regeneration and Its Clinical Implications

The liver is a major metabolic organ that performs a multitude of functions and has an incredible regenerative capacity (143). One of the earliest insights into the role of GC-C in the liver emerged from autoradiography studies by Krause and colleagues, who demonstrated the presence of a ST receptor in the adult opossum liver and documented that ST elicited a 7-fold increase in cGMP in the liver compared to a 30-fold increase in the duodenal glands (144). Subsequently, rodent studies revealed that GC-C expression in livers is temporally regulated, peaking during the perinatal period and remaining undetectable in adult livers (145, 146). Intriguingly, GC-C expression in adult livers was noticed in injury/regeneration models such as partial hepatectomy, intraperitoneal carbon tetrachloride (CCl4) injection, or subcutaneous turpentine oil injection (147). After partial hepatectomy, there was a significant and phasic increase in GC-C expression that peaked at 12h, reaching ileum levels, which declined at later time points.

In comparison, although exposure to CCl4 injection or turpentine oil injection showed similar upregulation of GC-C, it was much lower than in the case of partial hepatectomy (147). Independent studies validated these findings, demonstrating that GC-C protein levels were markedly elevated after partial hepatectomy, specifically in non-parenchymal cells and, to a lesser extent, in hepatocytes, lending credence to the potential role of GC-C in liver regeneration (148). During liver regeneration after CCl4 exposure, the expression of endogenous ligands (guanylin and uroguanylin) increases in coordination with GC-C expression (149).

Notably, these observations are consistent with a long-recognized view that increased guanylyl cyclase activity might play a role in liver development and regeneration (150, 151). The increase in GC-C expression, ST binding, and ST-stimulated cGMP accumulation in primary cultures of rat hepatocytes and a rat hepatoma cell line (H-35) after treatment with dexamethasone, alone or in combination with interleukin-6 (IL-6), all support the role of GC-C in the regulation of acute hepatic phase response (152). Although these results have given us an insight into a major extraintestinal role of GC-C, many questions remain. Specifically, what induces GC-C expression during liver regeneration and what are the consequences of increased cGMP remain unresolved. We note that HNF4A, the transcription factor that regulates GC-C expression, is well established to play a role in liver regeneration by regulating the acquisition of the fully differentiated phenotype (153, 154). Given the role of GC-C in regulating cellular differentiation (155), we believe increased intracellular concentrations of cGMP in replicating cells during regeneration or perinatal growth would be required to acquire the liver phenotype. Other regulatory functions for cGMP, such as cell-cell crosstalk, are also possible, based on observations of carrier-mediated cGMP release in hepatocytes (156).

The development of steatohepatitis in mice with a targeted disruption of GC-C lends some support to the role of GC-C in the hepatobiliary system (51). Perfusion experiments revealed that cell-permeable cGMP analogs stimulate fluid and ion secretion and increase bile acid–independent bile flow (157). In line with these findings, CFTR has been found in the apical domain of biliary epithelial cells, and its activation is cGMP dependent. A Cl^-^ secretory defect has been linked to liver disease in cystic fibrosis patients (158–160). In addition to CFTR, independent studies have reported the expression of GC-C and guanylin in the epithelial cells of the bile ducts of the liver and gallbladder (161, 162). The available evidence suggests that GC-C mediated CFTR regulation may modulate the ductular reaction and expansion of biliary epithelial cells in response to liver injuries, which has been linked to both repair and disease progression leading to inflammation and fibrosis (163). Recent studies have shown that regulation of the osmotic gradient, transcellular water transfer, and changes in the lumen volume play key roles in complex crypt-villus patterning and morphogenesis in the intestine (164). As previously outlined, GC-C activates CFTR and regulates ion secretion in the intestine; it is possible that it influences fluid and electrolyte flux in the liver and facilitates the coordination of epithelial morphogenesis during liver organogenesis. This role is consistent with the predominant expression of GC-C in the canalicular domain of the regenerating liver (147).

Together, these findings may point to a role for GC-C in the regeneration of the liver in response to injury, including the replenishment of hepatocytes, the reconstitution of damaged biliary epithelia, and the restoration of normal liver function. Studies in GC-C-null outbred mice documenting accelerated mortality by intraperitoneal injection of CCl4 provided corroborative evidence for the role of GC-C in effective recovery from acute toxic liver injury (149). Markers of liver injury, such as hepatocyte death, apoptosis, and areas of centrilobular necrosis, were exacerbated in CCl4 treated GC-C-null mice (149). Along the same lines, GC-C null mice and uroguanylin-null mice had increased apoptosis and slower recovery from non-lethal radiation injury to the intestine (165). However, it is unknown whether the protective mechanisms of GC-C signaling in the intestine and liver are similar, and more research is needed. Significantly, in the radiation injury mice model, cGMP supplementation reduced apoptosis and promoted intestine regeneration in GC-C null mice and uroguanylin-null mice (165). Translating these findings into potential therapeutic options, it would be worthwhile to investigate GC-C agonists or cell-permeable cGMP analogs as agents that can stimulate and accelerate regeneration in donor and transplanted livers and following hepatic resection.

### The Enigmatic Role of GC-C in the Pancreas and Its Relevance for Inflammation and Cancer

Transepithelial ion transport within the ductal system is critical to pancreatic function. Accumulating evidence now points to CFTR as a critical regulator of pancreatic transepithelial ion transport, which is consistent with its high expression in the pancreas and the fact that cystic fibrosis patients have prominent pancreatic pathology (166–168). Mechanistically, CFTR on the apical membrane is required for the pancreatic ductal epithelium to secrete a bicarbonate-rich fluid containing up to 140 mM ${\text{HCO}}_{3}^{-}$ (167). Given the evidence that GC-C/cGMP signaling regulates CFTR function in the intestine (6), it appears intuitive that GC-C and its ligands exist in the pancreas as potential local regulators of fluid and electrolyte secretion *via* a paracrine/luminocrine signaling pathway.

Indeed, cell-specific localization studies in the human and rat pancreas broadly support this possibility, with GC-C, uroguanylin, and guanylin found in the exocrine parenchyma confined to centroacinar cells and epithelial cells of the intercalated, intralobular, and interlobular ducts, but not in acinar cells or islet cells (169, 170). These results are consistent with independent studies demonstrating GC-C activity in the exocrine pancreas (171). Localization of GC-C in the pancreas parallels the expression of the transcription factor CDX2, which is known to regulate GC-C expression in the intestine (172, 173). Furthermore, guanylin is abundant in pancreatic juice, raising the intriguing possibility that guanylin released luminally into the pancreatic ducts may exert its function in the pancreas in a luminocrine fashion (174). Functional studies in human pancreatic duct cell lines expressing the CFTR wild-type or mutant (ΔF508) provided evidence that guanylin, *via* functional coupling proteins, acts as a specific regulator of pancreatic CFTR channel function. Guanylin increased Cl^-^ conductance in cells expressing wild-type CFTR to a similar extent as forskolin and ST but did not activate Cl^-^ conductance in cells expressing mutant CFTR (174).

An important caveat remains. Even though tissue distribution and cellular localization in human and rat pancreas indicated that GC-C was exclusively localized to the ductal system, it is not present in the endocrine pancreas (169, 170). This is consistent with functional studies demonstrating GC-C activity in exocrine pancreas (171) and guanylin-mediated CFTR activation in pancreatic ductal cells (174). However, GC-C expression in various mouse tissues produced strikingly opposite results (175). The authors report that in this experiment, GC-C was not detected in the entire pancreas but was present in isolated islets, leading them to conclude that its expression was most likely restricted to the islets (175). Furthermore, the expression of GC-C was higher in MIN6c4 cells, a subclone of the pancreatic β cell line, MIN6, derived from a mouse insulinoma, than in the parental cell line, correlated with the prolonged maintenance of insulin secretion in these cells as compared to parental cells (175). Depletion of GC-C in MIN6c4 cells resulted in decreased KCl-induced insulin secretion and content (175), which is consistent with previous reports that guanylin stimulated insulin secretion in a rat pancreatic cell line (176). Together, the studies above provide a complex picture of the localization and potential role of GC-C in the pancreas, which could be explained in part by the abundant plasticity within the normal endocrine and exocrine pancreas (177).

GC-C was among the highly upregulated genes in pancreatic acinar (AR42J) cells treated with caerulein as an *in vitro* model of acute pancreatitis (178, 179). Given the well-studied role of CFTR in pancreatitis (180), it is likely that GC-C also plays a role in the development and progression of pancreatitis; however, whether the induction of GC-C during pancreatitis serves a protective or detrimental function remains unresolved. In line with the *in vitro* pancreatitis model, GC-C expression was significantly upregulated in human chronic pancreatitis samples than in normal pancreatic tissue (103). Furthermore, when pancreatic cancer was compared to pancreatitis, the upregulation of GC-C was found to be even more significant in pancreatic cancer (103).

Notably, uroguanylin has been shown to inhibit pancreatic cancer cell proliferation, consistent with its role in regulating epithelial cell turnover *via* cGMP signaling (103). As with chronic pancreatitis and pancreatic cancer, prominent upregulation of GC-C (due to bile acid exposure) has been reported in Barret’s metaplasia and oesophageal adenocarcinoma, where it is thought to have a pro-tumorigenic effect by initiating lineage-addicted tumorigenesis *via* chronic suppression of the EGFR/AKT axis (181, 182). It is important to note that bile acids, specifically the reflux of bile acid into the pancreatic duct and to the epithelial cells or acinar cells, have been linked to pancreatitis, acinar to ductal metaplasia, and pancreatic cancer (183). Exposure to bile acids, like what has been reported in oesophageal cancer cells (181, 182), may underlie the upregulation of GC-C in pancreatitis and pancreatic cancer. More research is needed to determine whether GC-C plays a role in cellular reprogramming in the malignant pancreas and to assess the current state of evidence implicating both low and high GC-C to have context-dependent effects in this deadly disease.

### Potential Role of GC-C in Adipose Tissue Function and Fat Mobilization

Obesity is referred to as the silent endemic (184). It affects multiple organ systems and frequently leads to other co-morbid conditions referred to collectively as metabolic syndrome, which has drastic effects in increasing susceptibility to other diseases brought to prominence lately by the COVID-19 pandemic (185). As previously discussed, guanylins are anorexigenic peptides that regulate adiposity by activating brown adipose tissue and inhibiting energy storage in white adipose tissue (51, 131). Understanding the role of guanylins in central vs. peripheral regulation of whole-body energy balance could shed light on mechanisms underlying the pathogenesis of obesity and provide insight into developing therapeutic strategies for treating obesity and related diseases.

Given the contrasting findings of satiety response to central uroguanylin administration, it appears that the impact of uroguanylin is not solely mediated by central mechanisms (51, 134). One of the earliest insights into the potential role of the peripheral GC-C system in regulating adipocyte function emerged from transcriptomic analysis of rats fed a high-fat diet (HFD), which revealed high expression of guanylin and GC-C in mesenteric fat in lean rats that resisted dietary obesity, versus those that developed obesity (186). The mechanism and function of guanylin and GC-C induction in fat are unclear. Immunohistochemical analysis revealed that guanylin and GC-C are expressed by macrophages in the visceral fat depot, indicating that adipose tissue macrophages are the primary source of the increase in their expression seen in rats that resisted dietary obesity (186). This raises the intriguing question of whether GC-C/cGMP signaling plays a role in altering the inflammatory profile of adipose tissue macrophages.

Obesity is strongly linked to the induction of chronic inflammation, macrophage infiltration into adipose tissue, and metabolic dysfunction (187). Given the evidence that GC-C signaling can modulate inflammation in the intestine (13), it is tempting to speculate that guanylin derived from macrophages has anti-inflammatory autocrine and paracrine effects within adipose tissue. Consistent with this notion, double transgenic rats that overexpress both guanylin and GC-C in macrophages were resistant to a high-fat diet and escape insulin resistance (186). Mechanistically, GC-C/cGMP signaling in adipose tissue macrophages was shown to regulate mesenteric fat inflammation by inhibiting classical activation (M1) of macrophages in response to a high-fat diet (188). While genes involved in fat droplet formation were downregulated, those involved in fatty acid oxidation were upregulated in mesenteric fat in HFD-fed double transgenic rats compared to HFD-fed wild-type rats (186). Ex vivo studies revealed that rat and bovine adipocytes cocultured with guanylin and GC-C expressing macrophages showed significant inhibition of lipid accumulation, pointing to a role for GC-C/cGMP signaling in macrophages in the modulation of lipolytic energy mobilization by adipocytes (186, 189). The inhibition of lipid accumulation appears to be related to an increase in interleukin-15 secretion from guanylin and GC-C expressing macrophages, which inhibits fatty acid synthase in adipocytes and leads to obesity resistance (190).

As previously outlined, GC-C/cGMP signaling can increase intracellular levels of cAMP and cGMP, two secondary messengers known to be important in lipolysis (191, 192). In human adipose tissue, GC-C RNA and protein expression was detected in mature adipocytes as well as the stroma-vascular fraction (SVF), which contains a variety of cell types, including macrophages and vascular smooth muscle cells (123). Whereas circulating uroguanylin levels were reduced, GC-C expression appears to be upregulated in visceral adipocytes and various cell types of adipose tissue SVF in obese type 2 diabetes mellitus patients (123). Both guanylin and uroguanylin induced lipolysis in differentiated human omental adipocytes, as evidenced by phosphorylation of hormone-sensitive lipase at Ser563, an increase in fatty acid and glycerol release, and an upregulation of several lipolysis-related genes (123). In comparison, the potential role of peripheral GC-C/cGMP signaling in brown adipose tissue function is unknown. Although diet-induced thermogenesis and brown adipose tissue activation are reported to be GC-C dependent, GC-C expression is not detected in brown adipose tissue, indicating that these effects appear to be mediated through the hypothalamus-sympathetic nervous system-adipose tissue axis (19).

In summary, while the best-studied function of uroguanylin in obesity is in the central nervous system, where it stimulates anorexigenic pathways *via* GC-C receptor activation in the hypothalamus, there is growing evidence that it may also influence metabolic function peripherally, primarily *via* GC-C receptor in adipose tissue macrophages and adipocytes. Obesity, for example, induces GC-C in adipose tissue, where it may regulate multiple aspects of adipocyte biology. While GC-C induction in fat appears to correlate with insulin resistance and an increase in adipose tissue macrophages, activation of GC-C signaling in adipose tissue induces lipolysis and promotes fat mobilization, suggesting that GC-C in adipose tissue may have an anti-obesity role. This is supported by the fact that uroguanylin-deficient mice fed a high-fat diet are more obese and insulin-resistant. Surprisingly, GC-C knockout mice in this study had normal body weight, adiposity, and glucose tolerance (134). The origin of guanylin peptides and the mechanistic role of GC-C receptor activation in obese adipose tissue remains to be defined.

Given the disparities in body weight gain in whole body GC-C knockout mice fed a high-fat diet, it is possible that the central and peripheral effects of GC-C signaling on adipose tissue are context-dependent and may be influenced by other factors such as the outgrowth of obesity-inducing gut commensals (51, 134). Studies, for example, have shown that GC-C signaling is an immune mediator with both pro-inflammatory and anti-inflammatory properties (193, 194). In some studies, GC-C deletion in mice protects against experimental colitis and reduces inflammation (193), while in others, it exacerbates colitis by impairing gut barrier integrity (194). The gut microbiome appears to play a prominent role in the pathogenesis of colitis in GC-C null mice. More research is needed to determine whether GC-C and its ligands are expressed by adipose tissue during normal physiology and how they contribute to obesity-induced adipose tissue inflammation.

Furthermore, given the evidence of an anti-inflammatory effect of GC-C signaling on macrophage maturation in adipose tissue (188), it is important to determine how GC-C receptor activation inhibits M1 cytokine gene expression. Importantly, *in vivo* experiments with loss of GC-C expression, specifically in adipose tissue, are required to decipher the effects of the peripheral GC-C system. It is tempting to speculate that early induction of GC-C-mediated central and peripheral regulation of whole-body energy balance may control appetite and dampen the early inflammatory response to obesity. In this context, oral supplementation with linaclotide has recently been shown to stimulate brown fat thermogenesis and reduce body weight, providing a translational opportunity for reducing the risk of insulin resistance and type 2 diabetes mellitus (195).

### Physiology and Clinical Significance of Guanylins as Intestinal Natriuretic Hormones

The kidney is essential for maintaining extracellular fluid homeostasis, including sodium homeostasis, acid-base balance, volume regulation, blood pressure regulation, and glucose homeostasis (196). Despite the crucial role of sodium homeostasis in physiological processes, particularly blood pressure regulation, entero-renal mechanisms governing sodium excretion are not well defined. Nearly 50 years ago, pioneering studies by Lennane et al. found that oral administration of a NaCl load resulted in a greater natriuretic response than intravenous administration of the same load in both rabbits and humans (197, 198). This led to the hypothesis of intestinal natriuretic hormones, with guanylin and uroguanylin acting as potential candidates, that transmit signals from the GI tract to the kidney (53). How levels of these bioactive peptides dynamically change and orchestrate fluid-ion balance has been an active area of research.

Using a targeted gene disruption mouse model, Lorenz et al. demonstrated that uroguanylin was essential for regulating renal sodium excretion after enteral loading *via* the formation of a putative enterorenal axis for coordinating salt ingestion with natriuresis (53). This lends credence to the model that a high-salt diet causes prouroguanylin to be released from the intestinal epithelium into the circulation and delivered to the renal tubules, where it is processed to uroguanylin, resulting in increased sodium excretion in the urine (199, 200). Indeed, studies have shown that a high salt intake not only induces the expression of guanylins in the intestine and kidney but also increases uroguanylin secretion in the urine (37, 201–203). On the other hand, some studies demonstrating that a high salt diet induced uroguanylin expression primarily in the kidney rather than the intestine, led to the alternative model that diet-evoked uroguanylin signals originate in the kidney rather than circulating uroguanylin derived from the intestine (204, 205). Regardless, despite differing views on the origin of these peptides, mounting evidence suggests that the diuretic and natriuretic responses to guanylin peptides following high dietary salt intake play a key role in extracellular fluid homeostasis. A thorough understanding of how these bioactive peptides affect salt and water balance may provide valuable insights into the development of novel therapeutic options.

Uroguanylin knockout mice develop hypertension primarily because of renal salt handling deficits, implying that the gene could be a promising candidate for essential hypertension. This multifactorial disorder is a significant risk factor for death from cardiovascular and cerebrovascular events (53). Indeed, a haplotype-based case-control study found an association between uroguanylin and essential hypertension. However, more research is needed to identify and characterize susceptibility mutations in the *GUCA2B* gene for essential hypertension (206). Given the growing trend of disorders associated with dysregulated fluid-ion balance and the associated morbidity and mortality, it is important to draw insights from adaptive responses to volume overload to design and develop appropriate therapeutic approaches. One can speculate that upregulation of circulating uroguanylin levels observed during pathophysiological states of sodium retention and blunted volume expansion natriuresis, such as congestive heart failure and nephrotic syndrome, may serve as a compensatory and/or adaptive response, as well as a prognostic indicator of edematous pathophysiological states (207, 208). The converse is also true. The guanylin/uroguanylin signaling pathway is downregulated in the intestine as an adaptive response to salt restriction (201, 209).

Binding of guanylins to GC-C is necessary for cGMP production. As such, GC-C is considered a bonafide receptor of ST and endogenous guanylin peptides (6, 7). Early studies in the opossum and rat kidneys predicted the presence of a functional receptor for *E. coli* ST (210, 211). Subsequent research on endogenous peptides that resemble ST, uroguanylin, and guanylin, which were originally isolated from urine and intestine, respectively, revealed that they promote natriuresis, kaliuresis, and diuresis in perfused rat kidneys (8, 27, 28). Whether binding of guanylins to GC-C is required for mediating these effects in the kidney is not clear. Still, data from several studies suggest that renal effects of these peptides are mediated by signaling independent of GC-C (212, 213). For instance, although uroguanylin treatment increased renal sodium excretion in mice, it did so even when the receptor GC-C was deleted, demonstrating that additional pathways are required to specify the renal function of guanylin hormones (214). Previous research into the expression and localization of GC-C in the kidney has been limited because most studies rely on detecting transcript levels (53, 212, 215).

In summary, early studies encouraged the notion that the kidney also expresses GC-C and that the signals manifested by binding of guanylin peptides would result in increased excretion of salt and water in the urine, thereby regulating overall fluid-ion homeostasis. More recent research has undermined this notion, revealing that the guanylin peptides function *via* an unknown GC-C independent mechanism. Pertussis toxin-sensitive G protein-dependent and phospholipase A2-dependent signaling have been proposed as potential cGMP- and GC-C-independent mechanisms of action for guanylin peptides in kidney (9, 213). Physiologically, guanylin and uroguanylin-regulated phospholipase A2-dependent signaling has been reported to modulate K^+^ conductance in cortical collecting ducts, thereby changing the driving force for Na^+^ and water reabsorption (213). On the other hand, cGMP-dependent mechanisms of uroguanylin action in renal tubules have also been reported, including modulation of activities of Na^+^/K^+^- and H^+^-ATPases as well as NHE3 (216–218). An important agenda for future research is clarifying the signal transduction pathways that mediate the responses of guanylin peptides and dictate when and how the kidney can retain sodium and water or lose them in urine.

### Potential Role of GC-C/cGMP in the Synergistic Interaction of Circadian Rhythmicity and Feeding Behavior

Homeostatic states such as the biological clock and metabolism are intertwined. For example, orexins, the neuropeptides that regulate feeding, are well-known essential regulators of sleep/wake cycles and the circadian clock (219). Thus, the feeding cycle can entrain the circadian cycle; however, the neuro-molecular mechanism by which feeding regulates the circadian rhythm is not fully defined. The hypothalamic suprachiasmatic nucleus (SCN) controls the human biological clock (220). The circadian rhythm regulates various mechanisms in the human body, like blood pressure (221) and hormone secretion (222, 223). Some of the hormones regulated in the circadian fashion include insulin, leptin, ghrelin, and adiponectin; these are partially controlled by feeding behavior as well (222).

Further, the anatomical connection from the eye to the SCN is the ‘retinohypothalamic tract.’ This tract enables the adjustment of circadian rhythm to the dark-light cycle of the environment. Rats with SCN lesions lost the circadian regulation of corticosterone secretion (224). The body clock is regulated by genes initially identified in Drosophila, with the *Per* gene playing a negative role and the *Clock* gene playing a positive role (225).

Cyclic GMP is the major signaling molecule that regulates the biological clock *via* PKGII in the SCN, modulating circadian rhythms and synchronization to the day/night cycle (226, 227). Indeed, research suggests that sildenafil may be useful in treating circadian adaptation to environmental changes (227). PKGII is a master regulator of the circadian clock, which drives many physiological, biochemical, and behavioral rhythms, and is important in modulating the timing and quality of the sleep-wake cycle (226). Importantly, mice lacking PKGII cannot reset the circadian clock, despite having normal retinal function (226). Mechanistically, PKGII appears to control the light-induced modulation of Period 1 and 2 genes reciprocally, influencing the direction of phase shifts (advances or delays) of the clock (226). However, to date, the upstream hormone-guanylyl cyclase system that elicits cGMP production and regulates PKGII activity in SCN is unknown. Because GC-C is expressed in the hypothalamus and uroguanylin-mediated GC-C activation in the hypothalamus has been implicated in the control of appetite and energy expenditure (51), it is tempting to speculate that hypothalamic GC-C/cGMP signaling regulates PKGII signaling and influences the adjustment of the biological clock and coordinates synergistic interaction between the clock and feeding behavior. Further studies are needed to substantiate this hypothesis.

Studies have shown that the blood pressure of normotensive Wistar rats is rhythmically coordinated with soluble guanylyl cyclase levels in aortic tissues (221). Cyclic GMP plays a role in circadian entrainment in rats, proven by the administration of exogenous cGMP in both day and night conditions. Daytime exogenous cGMP treatment induced phase advances in the circadian rhythm of rats, as monitored by electrical stimulation from the SCN of hypothalamic sections maintained in a perfusion buffer. The circadian rhythm remained unaltered with similar treatment at night (228). Further, the pharmacological inhibition of PKG was found to prevent light-induced phase changes in the circadian rhythm of hamsters (229).

Circadian rhythm influences both the small and large intestine in mice (230). Apoptosis and development of clonogenic cells in the intestinal crypts of mice normalized to the circadian variation even when lighting conditions were reversed (231). In addition to hormonal modulation of circadian rhythm, dietary habits alter levels of DNA synthesis and proliferation in intestinal cells. These cycles, non-existent in the intestine at birth, arise after mice are weaned and develop a nocturnal feeding habit. Some parts of the gastrointestinal tract, like esophagus and rectum display a greater extent of circadian variation. The peaks of DNA synthesis varied by about 6-8 hours from the tongue to the anus, suggesting a dependence on the passage of food (225).

Oscillators and many clock-controlled processes have been observed in peripheral tissues of mammals (225, 232). What couples the peripheral oscillators, which are primarily regulated by food intake and metabolic changes, to the central pacemaker is not fully understood. The master clock, located in the hypothalamic SCN, is thought to entrain the phase of peripheral clocks *via* rhythmically secreted hormones. Feeding and associated metabolic changes could synergistically reinforce the circadian clock to attain entrainment. Changes in feeding behavior, like changes in photoperiod, may reset the circadian clock. Several genes involved in feeding, digestion, and absorption are controlled by a circadian clock (225, 232). In this context, it is important to note that the expression of guanylins and GC-C, and, by extrapolation, GC-C/cGMP signaling, exhibit circadian periodicity in the intestine of young rats (233). The transcript and protein levels of uroguanylin and guanylin were found to vary in light/dark cycles, with a peak during the dark photoperiod when the animals are actively feeding, with uroguanylin exhibiting greater variation than guanylin. Intriguingly, the cycle of uroguanylin and guanylin expression in the ileum and colon showed an anticipatory rise in the evening and peaked earlier than in the jejunum, suggesting that this regulation is circadian. GC-C, like its ligands, showed significant circadian variation at the mRNA level with upregulation (ileum > proximal colon > jejunum) during the dark photoperiod (233). The nocturnal increase in GC-C and its ligands may be essential to prepare the gastrointestinal tract for feeding by increasing fluid ion secretion required for mucus hydration and conferring lubricant properties to protect epithelial cells from mechanical stress during the passage of luminal contents and peristalsis waves. Additionally, given the existence of high-amplitude circadian rhythms in the expression of guanylin peptides, it is tempting to speculate that these hormones are secreted by the gut with circadian rhythm into the circulation and may act as entraining factors in the hypothalamus and thus mediate the synergistic bidirectional interaction between the master clock and peripheral clocks in the digestive system. This could also highlight the potential of FDA-approved uroguanylin mimetics, such as linaclotide, as chronobiotics, or agents that modify the characteristics of a circadian rhythm (phase, amplitude, or period).

## Learning Opportunities and Prospects

### Regulation of Endogenous GC-C Ligands

Despite ample evidence that dietary zinc regulates uroguanylin expression, the precise mechanism underlying this association has remained obscure (234). In 1996, Blanchard and Cousins discovered uroguanylin as one of several genes induced by a zinc-deficient diet in rats (235). Subsequently, the authors directly tested the hypothesis that zinc deficiency increased uroguanylin gene expression and proposed a role for increased uroguanylin expression in secretory diarrhea associated with zinc deficiency, as well as how supplemental zinc could correct secretory diarrhea (236, 237). Immunohistochemical studies revealed that in zinc-adequate rats, uroguanylin positive cells were concentrated at the tips of the villi of the duodenum and jejunum. In contrast, in zinc-deficient rats, the positive cells were dispersed throughout the villus (238). The upregulation of uroguanylin in response to zinc deficiency was exacerbated by an IL-1α-induced proinflammatory state (239).

Furthermore, dietary zinc deficiency increased the accumulation of uroguanylin derived from the systemic circulation in the rat kidney (240). Recent transcriptomic studies found that ‘cellular response to zinc ion’ was the only pathway significantly enriched in the ileum in patients with familial diarrhea syndrome due to an activating mutation (S840I) in GC-C compared to healthy controls, reinforcing the link between dietary zinc and GC-C signaling (241). Importantly, biochemical studies have demonstrated that zinc inhibits GC-C activity at concentrations comparable to those required to inhibit adenylyl cyclase (22). Recently, zinc has been shown to directly bind ST and inhibit GC-C activation and cGMP induction, providing an alternative mechanism for its inhibitory role in ETEC pathogenesis (242). These findings are consistent with previous studies that zinc-deficient diets allow ETEC to colonize the murine intestinal epithelium (243). Understanding the role of zinc in regulating uroguanylin expression and GC-C activity in fluid and ion homeostasis has important implications for developing interventions for diarrhoeal diseases and inflammatory bowel disease.

The therapeutic progress with GC-C agonists for the treatment of irritable bowel syndrome with constipation has prompted research into the regulation of endogenous guanylin peptides in different disease conditions (140). Recently, increased expression of guanylin peptides in lingual taste buds was reported, along with increased circulating levels, following sleeve gastrectomy, a weight-loss surgical procedure in which part of the stomach is removed, broadening the role of these hormones in food preference *via* the regulation of gustatory responses (244). Food intake raises circulating uroguanylin levels, but the stimulus for secretion is unknown (51). Although leptin, an adipocyte-derived hormone, has been linked to the nutritional status-based modulation of uroguanylin, direct supportive evidence is sparse (245). This link is particularly relevant to our understanding of the stimuli that inhibit postprandial secretion of uroguanylin into the circulation in obesity, which is evident in both human and animal models (123, 128–130). Notably, while genetic deletion of uroguanylin resulted in a significant decrease in guanylin expression, deletion of guanylin did not affect uroguanylin expression (52, 53). Recent studies attempting to decipher the mechanism of silencing guanylins in the context of colorectal cancer may provide important clues into their regulation in physiological conditions (15). Intriguingly, in some studies, uroguanylin levels in the circulation increased significantly with high dietary salt intake, but the hormone levels in the gut remained unchanged (204, 205). These observations offer insight into the coordination of uroguanylin levels in the intestine and plasma, raising new questions about the enterorenal endocrine axis and challenging the conventional view that diet-induced uroguanylin signals originate in the intestine.

### Structure-Function Analysis of GC-C Mutations

Ultimately, genetic testing in the clinic aims to help make clinical decisions. Structure-function analysis of patient mutations is the only way to differentiate pathogenic mutations from harmless polymorphisms. Although no atomic-level structures of GC-C is available, crystal structures of soluble guanylyl cyclases may serve as templates for modeling the cyclase domain, and available structures of protein kinases could serve as templates for the pseudokinase domain of GC-C (246). The causality of several patient mutations in GC-C has been established through functional studies (6). Most patient mutations in GC-C reported in the literature affect residues that are evolutionarily conserved, and therefore changes at these sites are predicted to cause dysregulated function (6, 16). What are the underlying structural changes that mediate a loss/gain of function of mutations at these residues, and do mutations in different domains produce proteins with altered conformation for loss/gain-of-function? Does binding of regulatory elements to GC-C alter the properties of a loss/gain-of-function mutation? These questions need to be answered if we are to understand GC-C biology and its relevance in human diseases.

Mapping mutations in the protein domains will also provide insight into the underlying pathogenic mechanism. For example, many reported activating GC-C mutations associated with congenital sodium diarrheas and IBD lie within the KHD, linker region, and GCD (10, 11, 113). On the other hand, GC-C missense mutations associated with meconium ileus have been localized to ECD, linker region, and GCD (12, 247, 248). This does not necessarily imply that other domains with no reported human mutations are merely inert linkers with a limited role in the structure and function of the receptor. For example, the juxtamembrane domain (JMD), one of the least studied domains, has no recorded human mutations in GC-C. However, this domain is predicted to serve essential regulatory roles such as facilitating dimerization, allosteric KHD modulation, and cyclase activity regulation. Evidence supporting this proposed role is the activating A488P mutation in JMD identified in the related receptor guanylyl cyclase B (GC-B) that was linked to skeletal overgrowth and showed enhanced basal and ligand-mediated production of cGMP (249).

These insights gained from patient mutations in GC-C have indicated that the GC-C activation is not a simple on-off switch where, in the absence of ligand, the cyclase domain remains inactive, and the active conformation is obtained upon ligand binding to the ECD and catalysis proceeds. Instead, disease mutations have shown how different domains can regulate the activity of the cyclase even in the absence of ligand binding and how minute changes in conformations and interactions within and between each domain can lead the catalytic site to active conformation.

### GC-C, Inflammation, and Cancer: Is There Increasing Overlap?

As previously discussed, activating mutations in GC-C are associated with IBD, supporting the observation that higher levels of mucosal cGMP are maintained in inflammatory states (10, 16, 250). An animal model of a gain-of-function mutation in GC-C confirmed these clinical observations (13). In contrast to expectations, IBD patients and an experimental colitis model showed downregulation of GC-C and its ligands in response to antecedent or concomitant inflammatory stimuli, implying that direct treatment with uroguanylin mimetic drugs can reduce inflammatory cytokines and play a protective role in inflammation (251). Circulating levels of GC-C ligand precursors were also reduced in Crohn’s disease patients (32). It is tentative to speculate that impaired gut barrier integrity and dysbiosis of the microbiome, for instance, depletion of *Lactobacillus* strains, associated with both GC-C gain and loss of function, may underpin their potential links to IBD (13, 14, 194).

In a murine model of intestinal inflammation caused by oral *S.* Typhimurium infection, transcription of GC-C, guanylin, and uroguanylin was found to be downregulated (14). Deletion of GC-C was found to exacerbate intestinal inflammation following *S.* Typhimurium infection, underscoring the importance of cGMP signaling in promoting recovery from intestinal inflammation (14). Inflammatory responses may reduce the expression of undiscovered transcriptional stimuli of guanylins.

Together, these could exemplify the Goldilocks scenario in biology, in which cellular functions rely on an optimal condition, with higher or lower extremes potentially impeding them. This paradigm could explain the pro-inflammatory effects associated with the gain or loss of GC-C mediated signaling *via* likely distinct mechanisms (**Figure 1**). This seemingly contradictory role for cyclic nucleotide signaling could add another layer of complexity to our understanding of IBD and the development of potential therapies.

Inflammation and tumorigenesis appear to have similar effects on GC-C regulation, most likely *via* partially overlapping mechanisms (15, 251). In the literature, as outlined earlier, several pathways linking silencing of the GC-C axis and colorectal cancer have been described. Another possibility is that the intestinal barrier disruption and dysbiosis associated with GC-C depletion may result in the infiltration of commensal bacteria and their molecular products, which drive chronic inflammation and malignant transformation, thereby contributing to the initiation of the development of invasive colon cancer (14, 194, 252). Clinical trials are underway to determine whether CRC is treatable with ligand supplementation. Data from clinical trials in healthy human volunteers revealed that oral supplementation of uroguanylin analogues linaclotide and dolcanatide did not persist to activate GC-C signaling in the distal rectum, emphasizing the need for developing colorectum-targeted drug delivery systems for specific delivery and improved bioavailability (81, 253). It would be interesting to see if reconstitution of GC-C signaling in colorectum improves barrier function and chronic inflammation and how these contribute to its role in preventing transformation (**Figure 1**).

Given the links between activating GC-C mutations and IBD in humans, as well as the supporting evidence from transgenic mice of GC-C activation, we cannot rule out that downregulation of GC-C and its ligands in IBD patients and in a mouse model of experimental colitis could be an adaptive mechanism of epithelial preservation (10, 13, 16). In this context, it is worth considering studies demonstrating that barrier loss in IBD is associated with a decrease in levels of occludin, a tight junction-associated protein, in patient biopsy specimens from IBD patients (254). Remarkably, occludin knockout mice are normal and, more importantly, have a lower severity of DSS-induced colitis due to apoptotic pathway blockage caused by caspase-3 downregulation. Thus, in the context of IBD, loss of occludin has been proposed as an adaptive mechanism to limit epithelial damage (254). Interestingly, GC-C knockout mice have been shown to downregulate occludins and other junction proteins, impairing intestinal barrier stability (194). GC-C knockout mice are also resistant to DSS-induced colitis. They have lower apoptosis, indicating a role for GC-C signaling as an essential mediator of IBD to promote mucosal homeostasis, though whether this can be therapeutically targeted requires further investigation (193). Taken together, it is tempting to speculate this adaptive mechanism as part of the pro-neoplastic effects of chronic intestinal inflammation, which would result in an accumulation of cells with downregulated GC-C signaling and associated cellular defects, facilitating neoplastic transformation.

Given the counterintuitive role of ectopic GC-C expression in Barret’s esophagus and esophageal adenocarcinoma (181, 182), it is critical to consider whether GC-C upregulation and chronic colitis may also promote tumorigenesis (**Figure 1**). Furthermore, patients with familial diarrhea syndrome due to S840I gain of function mutation in GC-C have increased susceptibility to chronic esophagitis (10); whether the pathologies are similar to those seen with GC-C induction in the esophagus due to bile acid exposure remains to be determined (181, 182). These links may also help us understand the potential role of GC-C upregulation along the transformation continuum in chronic pancreatitis and pancreatic adenocarcinoma (103). More research is needed to determine the precise mechanisms involved in the protumorigenic function of GC-C signaling. Given the reproducible association of GC-C activating mutations with IBD, it is important to consider that chronic activation of the GC-C pathway by ligand treatment may activate inflammasomes and trigger IBD-like immunopathology (255).

## Conclusions and Implications

The GC-C signaling pathway is widely regarded as one of the most important fluid and electrolyte balance regulators. The consequences of failing to balance intracellular cGMP levels are demonstrated by conditions that appear to be driven by dysregulation of fluid and ion balance and are associated with diseases such as IBD and colorectal cancer. These findings also raise new questions. It is unclear how fluid and electrolyte balance changes impact inflammation or tumorigenesis or whether the changes are due to effects independent of these roles. Identifying germline mutations that either activate or inactivate GC-C has facilitated progress in this field, leading to recognizing the role of the ion-microbiome-immune axis as an upstream driver and regulator in intestinal pathologies. These insights have collectively uncovered a critical role of intestinal fluid and electrolyte homeostasis in regulating microbiome composition and cross-talk with host immunity.

**Figure 5:**
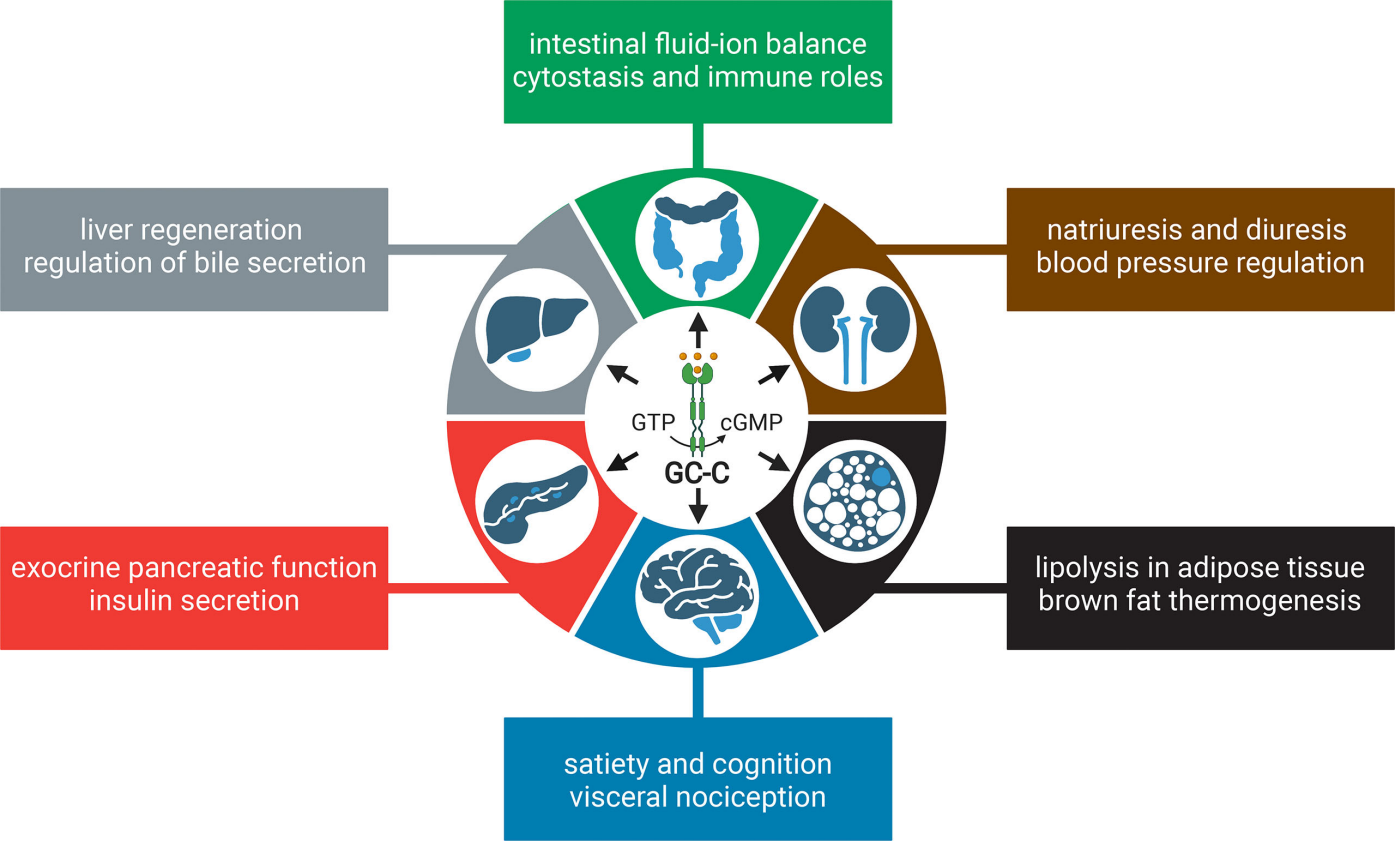
Summary of the multiple biological functions of GC-C/cGMP signaling in health and disease. The illustration depicts the role of GC-C/cGMP signaling in the intestine and extraintestinal tissues, with key functions highlighted. The figure was prepared using Biorender.

In recent years, research has focused on determining the precise role of GC-C signaling in human health and disease. It is now clear that GC-C and its ligands perform functions other than simply regulating fluid and electrolyte balance. This includes findings on GC-C coordinating appetite control and energy homeostasis, behavioral functions, and emerging role in pathologies such as obesity and metabolic syndrome. GC-C/cGMP signaling is increasingly recognized as a modulator of physiological processes in various extraintestinal tissues (**Figure 5**). Furthermore, the development of transgenic and knockout mice provides an option for further dissecting the role of GC-C and relating them to whole-animal physiology. More recently, small molecule inhibitors of GC-C have been developed as a targeted option for therapy in patients with activating GC-C mutations (256). Another promising approach would be to test the palette of drugs targeting kinases that could bind the KHD and inhibit the cyclase activity (257). Determination of the high-resolution structures of different domains of GC-C and structure-based design will expand on small molecules that can target GC-C and modulate its activity. Importantly, small molecules that target the catalytic or regulatory domains to promote enzymatic activity will create a new class of pharmaceuticals to activate GC-C independent of the ligand-mediated stimulation.

## Author Contributions

All authors contributed to writing and preparing the final versions of the manuscript.

## Funding

Support from the Department of Biotechnology, Government of India is acknowledged (BT/PR15216/COE/34/02/2017). SV is a JC Bose National Fellow (SB/S2/JCB-18/2013) and a Margdarshi Fellow supported by the Wellcome Trust DBT India Alliance (IA/M/16/502606). HP is an Early Career Fellow of the Wellcome Trust DBT India Alliance (IA/E/17/1/503665).

## Conflict of Interest

The authors declare that the research was conducted in the absence of any commercial or financial relationships that could be construed as a potential conflict of interest.

## Publisher’s Note

All claims expressed in this article are solely those of the authors and do not necessarily represent those of their affiliated organizations, or those of the publisher, the editors and the reviewers. Any product that may be evaluated in this article, or claim that may be made by its manufacturer, is not guaranteed or endorsed by the publisher.
